# Towards Urban Archaeo-Geophysics in Peru. The Case Study of Plaza de Armas in Cusco

**DOI:** 10.3390/s20102869

**Published:** 2020-05-19

**Authors:** Nicola Masini, Giovanni Leucci, David Vera, Maria Sileo, Antonio Pecci, Sayri Garcia, Ronald López, Henry Holguín, Rosa Lasaponara

**Affiliations:** 1 Istituto di Scienze del Patrimonio Culturale–CNR, Seat of Potenza, C.da S. Loya, 85050 Tito Scalo (PZ), Italy; nicola.masini@cnr.it (N.M.); maria.sileo@cnr.it (M.S.); antonio.pecci@unibas.it (A.P.); 2 Istituto di Scienze del Patrimonio Culturale–CNR, Seat of Lecce, Prov.le Lecce-Monteroni, 73100 Lecce, Italy; 3 Universidad Nacional de San Antonio Abad del Cusco, Av. de La Cultura 733, Cusco – Peru, Cusco 08000, Peru; david.vera@unsaac.edu.pe (D.V.); sayri.garcia@unsaac.edu.pe (S.G.); comandernod@gmail.com (R.L.); henryholguing@gmail.com (H.H.); 4 Dipartimento di Scienze Umane, Università degli Studi della Basilicata, Via Nazario Sauro, 85, 85100 Potenza, Italy; 5 Istituto di Metodologie di Analisi Ambientale–CNR, C.da S. Loya, 85050 Tito Scalo (PZ), Italy; rosa.lasaponara@cnr.it

**Keywords:** urban archaeology, urban geophysics, ground-penetrating radar, Cusco, Inca Empire

## Abstract

One of the most complex challenges of heritage sciences is the identification and protection of buried archaeological heritage in urban areas and the need to manage, maintain and inspect underground services. Archaeology and geophysics, used in an integrated way, provide an important contribution to open new perspectives in understanding both the history of cities and in helping the decision makers in planning and governing the urban development and management. The problems of identification and interpretation of geophysical features in urban subsoil make it necessary to develop ad hoc procedures to be implemented and validated in significant case studies. This paper deals with the results of an interdisciplinary project in Cusco (Peru), the capital of Inca Empire, where the georadar method was applied for the first time in the main square. The georadar method was successfully employed based on knowledge of the historical evolution of Cusco and the availability of archaeological records provided by some excavations nearby the study area. Starting from a model for the electromagnetic wave reflection from archaeological structures and pipes, georadar results were interpreted by means of comparative morphological analysis of high amplitude values observed from time slices with reflectors visualized in the radargrams.

## 1. Introduction

In the last decades of the 20th century, urban archaeology has developed in Europe and in North America in response to the rapid urban development and its impact on archaeological documentation. It also arises from the need to overcome chronological and disciplinary barriers in the field of archaeology which limits its effectiveness in answering the questions that the long history of an ancient city typically poses. Thus, urban archaeology represents a cognitive tool for a comprehensive and diachronic analysis of social, cultural, political events that occurred in ancient cities over time.

This is true in the case of archaeological ruins, and especially in the case of underground structures that often re-emerge fortuitously and traumatically, following excavations made for the construction of buildings or infrastructures. Therefore, in line with the compromise needed between the planning of city development and the preservation of cultural heritage (with particular reference to the conservation of buried and of unearthed vestiges), urban archaeology can provide methodological approach and a comprehensive interpretative analysis based on operational tools as geophysical methods. In particular, the understanding of the interface between urban modern settlements and ancient remains in the subsoil constitutes one of the most important challenges not only in archaeology but also in civil engineering and urban planning. To this aim, urban geophysics has a great growing potentiality as an operational tool useful for (i) improving planning and design of urban and infrastructure development and (ii) preserving cultural resources buried into the subsoil of historic cities, as in the case of Cusco—the object of analysis in this paper.

## 2. Urban Archaeo-Geophysics: Overview and Future Challenges

Nowadays, geophysical methods are successfully applied for addressing hydrogeological, civil engineering, environmental, and archaeological issues. In the latter case, these methods are generally more used in suburbs than in urban areas due to the complexity of urban subsoil, especially in cities with a long continuity of life. The presence of modern underground services affects and disturbs natural and artificial stratifications heavily, thus making the interpretation of geophysical investigations very complex and difficult.

To this regard, “the London case” (1995) was emblematic. The ground-penetrating radar (GPR) investigations performed by [1] in the subsoil of central London completely failed in the light of the data produced by the subsequent excavations. These unsuccessful results suggested a new approach in the GPR data interpretation based on the identification of a sort of ‘GPR fingerprint’ for a variety of archaeological deposits and features [1]. Nevertheless, “the London case” adversely impacted the development of geophysics for urban archaeology together with two additional reasons: (1) the limits of the GPR data processing tools at that time (before 2000s); (2) and, above all, the low demand of geophysical investigations in urban area.

More recently, as in the cases [2,3,4,5,6,7,8], successful investigations were conducted. In detail, the subsoil of the ancient city of Potsdam, using the electric, magnetic, electromagnetic methods and GPR methods, which allowed the detection of (i) two ancient ditches around the original town, (ii) the foundations of the city castle, and (iii) the main church was studied by [2]. The subsoil of Kaifeng (Henan, China) nearby a gate of the city walls using GPR and electrical resistivity tomography (ERT) was studied by [3]; thus discovering, after excavation, archaeological remains at different depths dated to different historical periods. The potential of multifrequency electromagnetic induction in archaeology in two heritage sites Han Hangu Pass and Xishan Yang was evaluated by [4], both in urban areas south of the Yellow River. Integrated archaeological records, with historical and cartographic sources, field surveys, remote sensing, geo-magnetometry and geophysical techniques were used by [5]. The aim was to detecting buried remains and reconstructing the landscape of ancient Ravenna, when (5th century A.D.) it was the capital city of the Western Roman Empire. The feasibility of GPR methodology in an urban area for the location of buried structures of archaeological interest was shown by [6]. Shallow cavities in the historical center of Matera using GPR and microwave tomography were detected by [7]. 2D-ERT survey for the identification of buried historical structures beneath the Plaza of Santo Domingo in Mexico City was used by [8]. Results from GPR survey carried out in the historical center of Lecce that have proven very important for the knowledge of buried archaeological evidence were show in [9], in particular, regarding the matter of the Messapian necropolis. The subsoil of San Benedetto del Tronto to identify the presence of natural voids and ancient anthropic underground structures using ERT and GPR was successfully investigated [10]. 

This brief overview highlights both the potentiality and complexity of urban geophysics along with the needs and importance to give a strong impulse to geophysical investigations in urban areas necessary both to (i) support objective decisions in urban planning and (ii) protect archaeological heritage preserved up to now into the subsoil of cities with a long history.

This paper deals with the GPR investigation conducted to explore the archaeologically sensitive areas located into the subsoil of Cusco, the ancient capital city of the Incas Empire. After the Spanish conquest, Cusco was partially rebuilt on the foundations and structures of the pre-existing Inca buildings and monuments.

The investigations based on the GPR prospection were conducted in the context of a bilateral project between Universidad Nacional de San Antonio Abad del Cusco (UNSAAC) and the National Research Council of Italy (CNR). The research project was aimed at assessing the GPR capability for the detection of hidden structures of archaeological interest in the Cusco urban area. These preliminary investigations were carried out to produce an archaeo-geophysical map to support the management and maintenance of underground services, in the respect of the archaeological vestiges.

**Figure 1:**
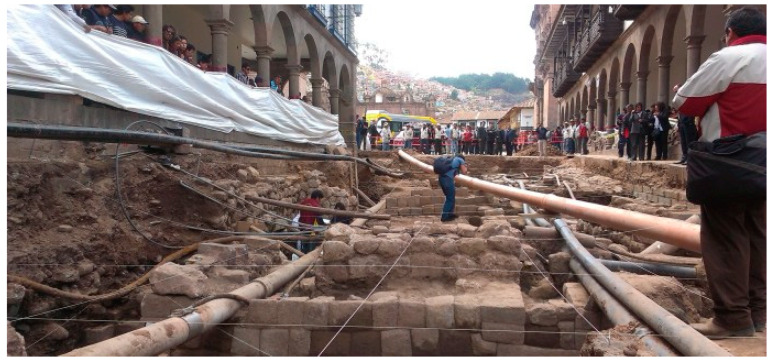
Archaeological find in Calle Mantas, one of the entrance ways to Plaza de Armas (courtesy of ANDINA/Percy Hurtado Santillán).

The idea of the project was born after a fortuitous discovery of Inca-era wall structures during the excavation made for subservice maintenance. Along the roads leading to Plaza de Armas in 2014 (Figure 1), these excavations unearthed walls of thickness range from 50 to 90 cm. They are composed of finely dressed granite blocks (typical of Inca ashlar masonry), are laid in both regular and irregular courses with an internal core of rubble masonry, and are composed of uneven stones set in mortar. In spite of the building features of the walls attributable to the Inca period, however, the excavations also brought to light pre-Inca ceramic fragments [11]. Finally, the excavation put also in evidence underground services (pipes of the aqueduct and sewerage), with most of them above the head of the walls (Figure 1) and others intrusive in nature, crossing the walls.

In the context of those found in the excavation at Calle Mantas, which can reasonably be representative of the other areas under investigation, the elaboration and the interpretation of GPR outputs are very complex due to the need to discriminate signals, i.e., reflectors linked to masonry structures of archaeological interest from reflectors referable to pipes or structures linked to water and sewerage network.

**Figure 2:**
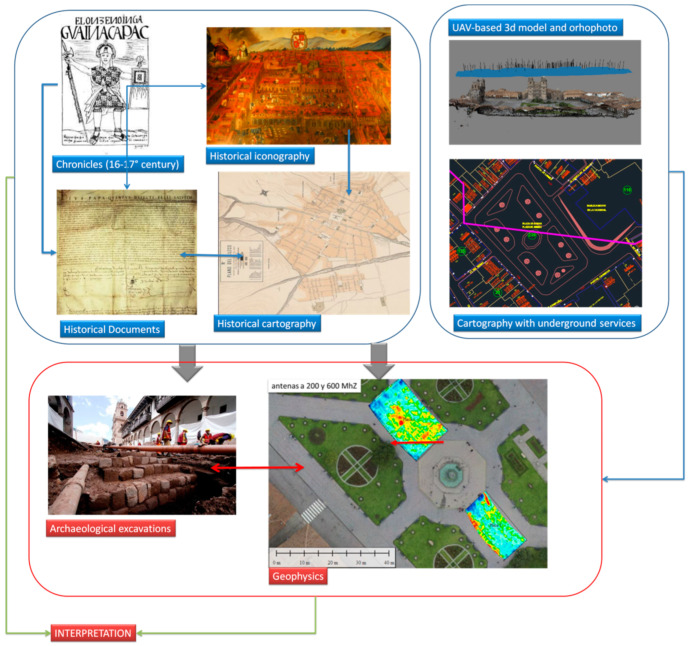
Methodological approach.

Considering that the GPR is the only geophysical method applicable in Cusco, the interpretation was made using (i) comparative observation of GPR time-slice amplitude maps with radargrams, appropriately geo-referenced on digital cartography along with (ii) the integration of GPR outputs with ancillary datasets made up of an urban map with underground services and information provided by the historical data sources and archaeological records (Figure 2).

## 3. Methodological Approach and Study Area

### 3.1. Methodological Approach

The methodological approach (Figure 2) was based on the integration of a heterogeneous dataset including information provided from historical sources, urban maps with underground services, UAV based surveys, and results from GPR based analysis.

**Figure 3:**
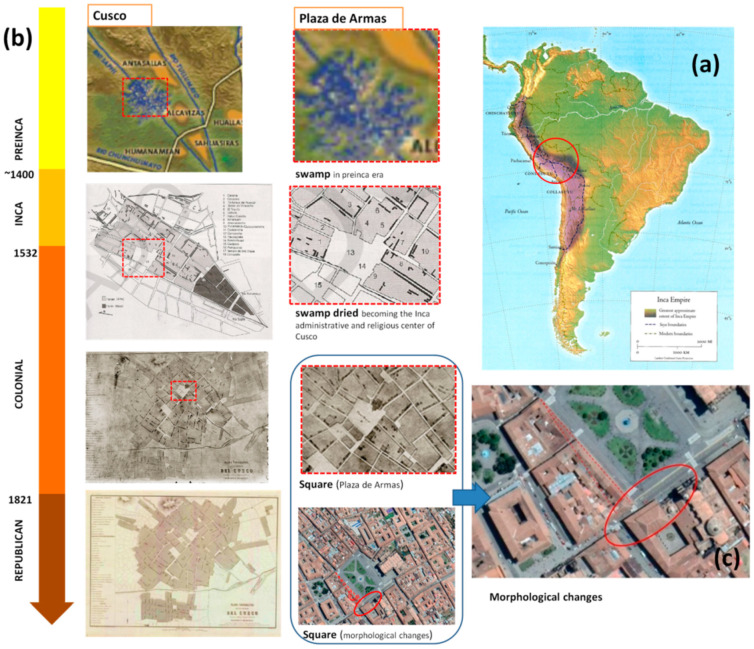
(**a**) Cusco capital of Inca Empire; (**b**) historical development of Cusco and Plaza de Armas; (**c**) detail of Plaza de Armas with indication of some morphological changes of the main square from the Colonial to Republican era.

The historical sources, including the narrative ones (the so-called Cronicas), published sources, historical iconography and cartography and ethnohistorical sources allowed the reconstruction of the historical development of Cusco at an urban scale, as shown in Figure 3.

In particular, the narrative sources [12,13,14] allowed us to define the main phase of the Cusco landscape evolution from the last decades of Inca period (1500–1542) to the first centuries of the Viceroyalty of Peru (1542–first half of the 18th century). Provided the most reliable information on the Inca Empire and, of course (being recorded coeval to the events), the first decades of Spanish rule (Figure 3) [12] This allowed us to understand the urban landscape and the architecture during the transition between Inca and Spanish rule, and, therefore, to capture continuity and discontinuity. In particular, palaces, churches and monasteries were largely constructed on the foundations of the Inca structures, for example, the Tempe of Sun (also known in Quechua as “Qoricancha”) was demolished by the Spanish colonists to build the Convent of Santo Domingo, and the Cathedral in the northern area of the main square was built following the destruction of “Kiswarkancha”, incorporating the Inca stonework into the structure of the new architecture.

The comparative analysis of the available historical maps of Cusco made it possible to identify changes in the building fabric, including some in the main square, and suggested areas to be investigated by GPR.

The urban map of Cusco with underground services was very useful in the interpretation of georadar maps. In particular, it helped us to reduce the errors in the interpretation of reflectors, differentiating those caused by wall structures from those related to aqueduct and sewer pipes.

**Figure 4:**
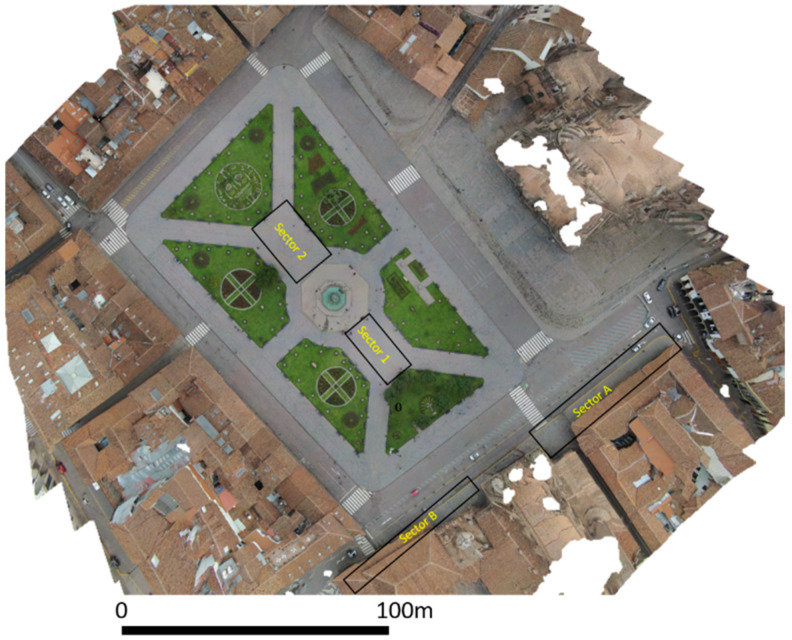
Plaza de Armas and the ground-penetrating radar (GPR) surveyed areas.

The urban maps have been integrated by a more detailed orthophoto of Plaza de Armas obtained by processing RGB images taken from drone using structure from motion, as shown in Figure 4.

Finally, regarding the GPR data analysis, beginning from a model to understand the nature of the electromagnetic (EM) wave reflection from archaeological structures and pipes (as reasonably as they are detected; see Figure 1), the GPR result interpretation is performed by means of comparative morphological analysis of high amplitude values observed from time slices with reflectors observed from the radargrams. From their matching, a number of features are extracted and analyzed by means of three-dimensional amplitude visualization.

### 3.2. Study Area: Cusco and Plaza de Armas over Time

The space occupied by the city of Cusco and the territory in which it sits is the result of the continuous transformations completed over time by the human groups that have inhabited it. Through the information collected from the archaeological records, in particular the 16th–17th century Spanish chronicles and historical studies, it is known that the city of Cusco is the result of six or more centuries of urban history that can be summarized into five successive historical phases the: (i) Killke settlement (pre-Inca age), (ii) Inca, (iii) colonial, (iv) republican, and (v) contemporary cities. In this long process, buildings and streets underwent different changes, destruction and reconstructions that shaped the current historical city. In particular, pre-Inca settlements (before the 15th century), occupied the area called Acamama, later known by the name of Cusco, divided into four neighborhoods. Cusco developed along two rivers, the Rio Salphy and Rio Tullumayo which were later channelized by Inca in the Pachacutec age, during which the division of neighborhoods were changed. The organization of the space, however, continued to be divided in four parts, including the center where the elite lived and the continuous suburbs of the center. In its surroundings, some satellite neighborhoods were settled. Regarding the main square, as we know from the report by Garcilaso de la Vega [13] and archaeological evidence [11], the Inca walls were encompassed on the facades of the Plaza de Armas, thus confirming that the limits of the current Plaza de Armas coincide, for the most part, with those of the old Awkaypata square. Only the cathedral and the houses built on the Saphi River have altered their old layout. The comparison of colonial maps with later maps put also in evidence some changes of the main square in the southeast, as shown in Figure 3. The southeast corner of Plaza de Armas is one of the town areas selected to be the object of GPR-based analysis, whose results will herein be shown and discussed in this paper.

### 3.3. Research Aims

The aims of this study are twofold: one technological and scientific, the second archaeological.

Aim 1: Evaluation of the potential of GPR in imaging archaeological features in a complex stratigraphy, as can typically be found in an urban area such as Cusco, distinguishing said features from other potential reflectors such as pipes, infrastructures etc. Hence, the archaeo-geophysics challenge as discussed in Section 2.

Aim 2: The current debate in the field of Inca archaeology in the city and region of Cusco regards understanding the consistency of previous phases of the Inca. In other words, to what extent is there continuity and discontinuity between the Inca and pre-Inca phases? Additionally, where can this pre-Inca phase can be found? Thus, GPR prospection was also aimed at identifying further anthropogenic layers, exploiting the available archaeological records unearthed by archaeologists during the excavations in Calle Manta, as shown in Figure 1.

## 4. GPR-Based Investigations of Cusco

Several geophysical methods are applied in urban areas with the aim to find buried archaeological structures [15,16]. These allow one to obtain high-resolution images of the subsurface. In this study, we used the GPR method which is based on the detection of variations in the electromagnetic (EM) properties of the subsoil, and subsequently uses these data to identify archaeological features and distinguish them from subsurface pipes. The GPR prospection was carried out with an IDS Hi Mod system with the 200–600 MHz dual-band antenna. Data were acquired in continuous mode along 0.5-m-spaced survey lines in both x and y directions, using 512 samples per trace, 70 ns two-way time (TWT) for 600 MHz antenna and 130 ns TWT for 200 MHz antenna, and a manual time-varying gain function.

The transect spacing was 0.5 m in the x and y directions. Assuming the suggestions in [17], the transect spacing should be 0.25 m with only parallel profiles. In theory, this should preclude the use of time slicing on these data. The effective target area is measured by the radius of the Fresnel zone (Fz) or the footprint area [6,15]. Table 1 shows the Fresnel zones at several depth related to the 200 MHz and 600 MHz antennas.

As the anomalies lie within 2 m of the present-day surface and the profiles were acquired in x and y directions, the resolution of time-slices will be near the optimum. Furthermore pioneering work on GPR time slicing (e.g., [15]) has shown that it is possible to obtain useful pattering from time-slices constructed from relatively widely spaced two-dimensional profiles, despite reduced resolution capacity. This is also because for the surveyed area, the expected dimensions of archaeological features range from 0.8 to more than 2 m. Recently, the distance between the GPR transect useful for obtaining excellent results has been discussed with several application examples in [16].

**Figure 5:**
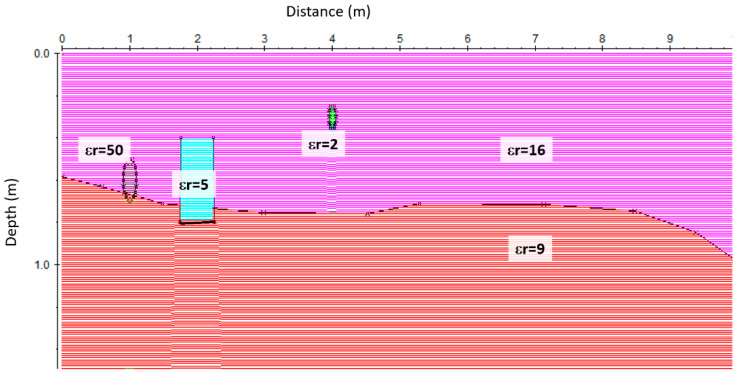
Forward model: two homogeneous layers with dielectric constants of 16 and 9 were modelled for the terrain and more compact layer, respectively. In the terrain, two pipes with different dielectric constant were inserted with εr = 50 and εr = 2. A wall feature, with a dielectric constant of 5, was placed at the contact between the two modelled layers.

The data were subsequently processed using standard two-dimensional processing techniques by means of the GPR-Slice Version 7.0 software [18]. The processing flow-chart consists of the following steps: (i) Frequency filtering. (ii) Manual gain, to adjust the acquisition gain function and enhance the visibility of deeper anomalies. (iii) Customized background removal to attenuate the horizontal banding in the deeper part of the sections (ringing), performed by subtracting in different time ranges a ‘local’ average noise trace estimated from suitably selected time–distance windows with low signal content (this local subtraction procedure was necessary to avoid artefacts created by the classic subtraction of a ‘global’ average trace estimated from the entire section, due to the presence of zones with a very strong signal). (iv) Estimation of the 2D electromagnetic wave velocity. EM wave velocity was determined from the reflection profiles acquired in continuous mode, using the characteristic hyperbolic shape of reflection from a point source [19]. This is a very common method of velocity estimation and it is based on the phenomenon that a small object reflects EM waves in almost every direction. (v) 2D Kirchhoff migration. (vi) Depth axis conversion using a constant average velocity value of 0.07 m/ns. The migrated data were subsequently merged together into three-dimensional volumes and visualized in various ways in order to enhance the spatial correlations of anomalies of interest. In order to understand the nature of the EM wave reflection from an archaeological structure and a pipe, a 2D model was produced using the reflex software [20]. This program allows the user to generate a synthetic model of what might be expected using known properties of the ground and the geometry of underground features [20]. A model of a stratified subsoil with the presence of pipes and walls was assumed, as shown in Figure 5. Two homogeneous layers with a dielectric constant of 16 and 9 were modelled for the surface terrain and more compact layer, respectively. In the terrain, two pipes with different dielectric constant were inserted. The first one simulated a water-filled pipe (εr = 50), and the second a metal cable-filled pipe (εr = 2). A wall feature, with a dielectric constant of 5, was placed at the contact between the two modelled layers.

**Figure 6:**
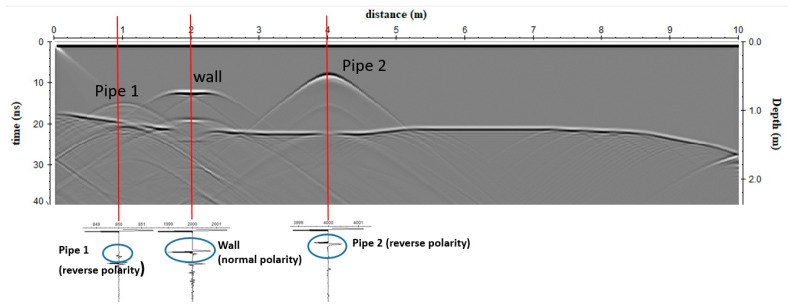
Forward model: radar section with the shown polarity of the electromagnetic (EM) wave reflected from pipe 1, pipe 2 and the wall.

The synthetic reflections (Figure 6) demonstrated that the pipes generate reflections when energy is intersected in the same pipe space, as was expected. Other reflection events were generated at the contact between soil 1 and 2 and from the simulated wall. When radar energy is reflected from a buried interface where the EM wave velocity decreases, the polarity of the reflected wave will be the same as the direct wave generated from the transmitting antenna [16,21]. This is the normal case in most ground conditions, and therefore, most reflections are recorded as normally polarized sine waves. Usually, as radar energy moves deeper into the ground, moisture retention increases and EM wave velocity decreases. If a drastic increase in velocity occurs at a boundary, for example, when waves enter a pipe space, a reflection will be generated that is visible in traces as a reversed polarity sine wave [16,21].

**Figure 7:**
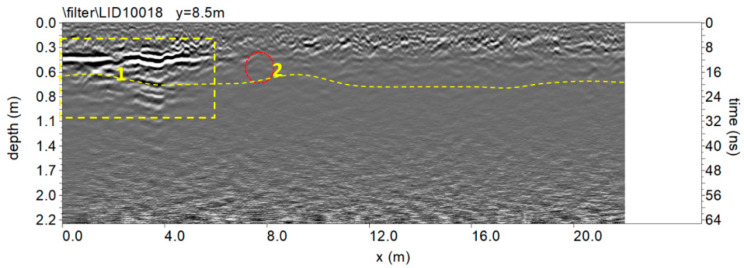
The 600 MHz processed radar section.

The investigated areas were some sectors of Plaza de Armas (named sectors 1 and 2, respectively) and two arcades on the south eastern side of Plaza de Armas (named sector A and sector B) (Figure 7). In Plaza de Armas, the two sectors are located at the southeast and the northwest of the octagonal fountain, respectively. The two areas, respectively 10 × 23 m and 15 × 25 m, were investigated in both orthogonal directions, with profiles spacing of 0.5 m. At the southern side of Plaza de Armas, two rectangular sectors were investigated. In this area, some pipes are expected to be detected as shown from some maps of Cusco. In regard to the arcades, sector A was 4 × 45 m while sector B was 2 × 35 m, which were investigated only along the longitudinal directions with profile spacings of 0.5 m.

## 5. Results and Discussion

### 5.1. Plaza de Armas Sector 1

Figure 7 shows the trace reflections and illustrates the difference between reflections (normal polarity) generated from the probable archaeological features (yellow box labelled 1) and the probable pipe (red circle labelled 2).The change of polarity to a reversed polarity reflection at this feature is confirmation that this feature is likely a pipe. The reflection from the slightly undulating (yellow dashed continuous line) exhibits normal polarity. This reflection could be related to a probable ancient living surface.

A way of obtaining visually useful maps for understanding the distribution plan of reflection amplitudes within specific time intervals is the creation of horizontal time slices. These are maps on which the reflection amplitudes have been projected at a specified time (or depth), with a selected time interval [22]. In a graphic method developed by [23], termed “overlay analysis”, the strongest and weakest reflectors at the depth of each slice are assigned specific colors. This technique allows for the linkage of structures buried at different depths. This represents an improvement in imaging because subtle features that are indistinguishable on radargrams can be seen and interpreted more easily. In the present work the time-slice technique has been used to display the amplitude variations within consecutive time windows of the width Δt = 5 ns, which corresponds to a soil thickness of about 0.15 m if an EM wave velocity of 0.07 m/ns is used.

**Figure 8:**
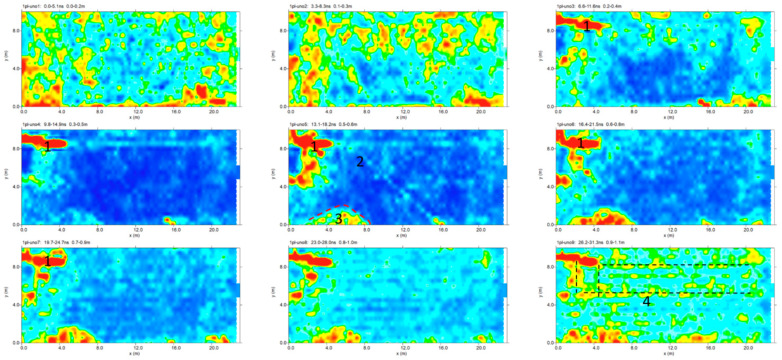
Plaza de Armas: sector 1: time slices in which some alignments are highlighted.

The time slices show the normalized amplitude using a range defined by blue as zero and red as 1. The time slices for the sector 1 are showed in Figure 8.

In the slices ranging from 0.2 to 1.1 m in depth, relatively high-amplitude alignments are clearly visible as the anomalies evidenced in the radargram (Figure 7). In the time slices in Figure 8 ranging from 0.5 to 0.6 m in depth, the weak oblique alignment (labelled 2) seems to show a probable pipe. The deeper slices (0.5–1.1 m) show other high amplitude alignments (labelled 3).

**Figure 9:**
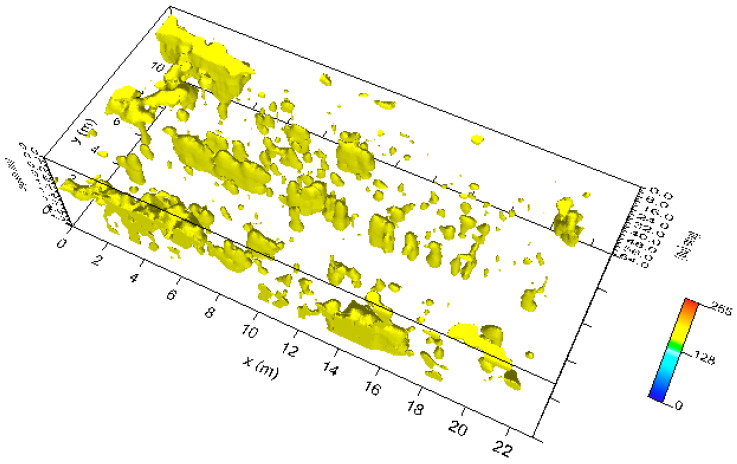
Plaza de Armas: sector 1: three-dimensional amplitude.

Moreover, the highest amplitudes were rendered into an isosurface [21,24,25]. Three-dimensional amplitude isosurface rendering displays amplitudes of equal value in the GPR study volume. Shading is usually used to illuminate these surfaces, giving the appearance of real archaeological structures. In this case, the threshold calibration is a very delicate task in order to obtain useful results. Figure 9 shows the three-dimensional amplitude isosurface using the 54% threshold.

In sector 1, the 200 MHz antenna results do not show results other than those obtained with the 600 MHz antenna.

### 5.2. Plaza de Armas Sector 2

**Figure 10:**
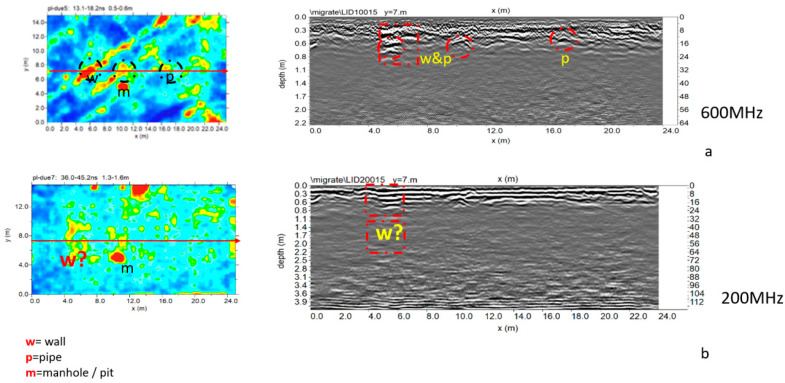
Plaza de Armas: sector 2: (**a**) 600 MHz antenna; processed radar section and time slice (0.5–0.6 m depth); (**b**) 200 MHz antenna; processed radar section and time slice (1.3–1.6 m depth).

Figure 10 shows the trace reflections and the corresponding time slices. In this case, it was also possible to illustrate the difference between reflections (normal polarity) generated from the probable archaeological features (red boxes labelled w) and the probable pipe (red box labelled P). The high amplitude anomaly labelled m is due to the presence of a pit in the surface. The presence of pipes is confirmed by a map of underground services in Cusco.

**Figure 11:**
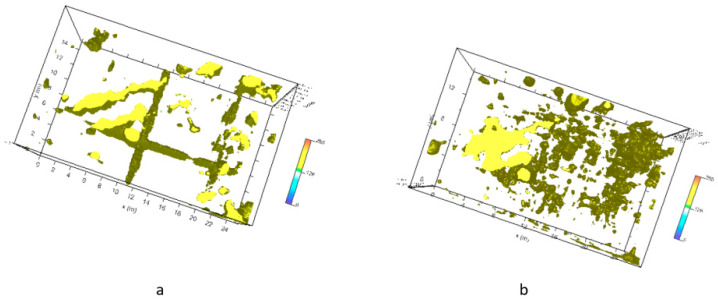
Plaza de Armas: sector 2: three-dimensional amplitude: (**a**) 600 MHz antenna; (**b**) 200 MHz antenna.

Figure 11 shows the three-dimensional amplitude isosurface using the 50% threshold.

### 5.3. Plaza de Armas Sector A

**Figure 12:**
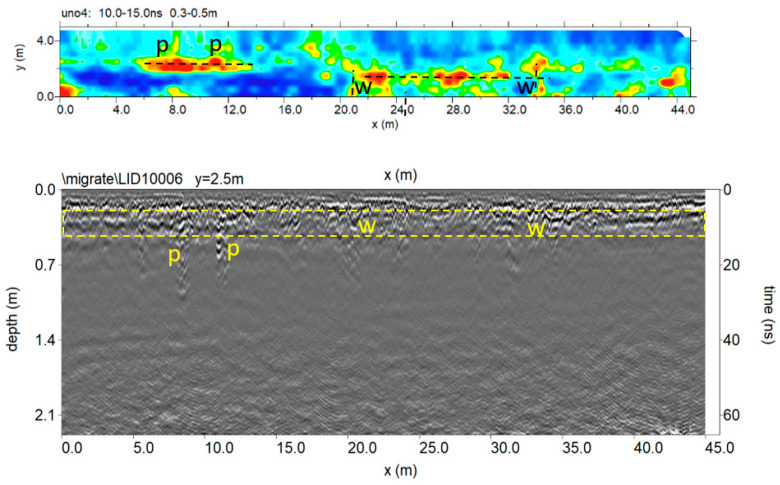
The 600 MHz processed radar section and time slice.

Figure 12 shows the trace reflections and time slice (0.3–0.5 m depth) for 600 MHz antenna. In this case, it was also possible to underline in the shallow subsurface of the probable archaeological features and the pipes.

**Figure 13:**
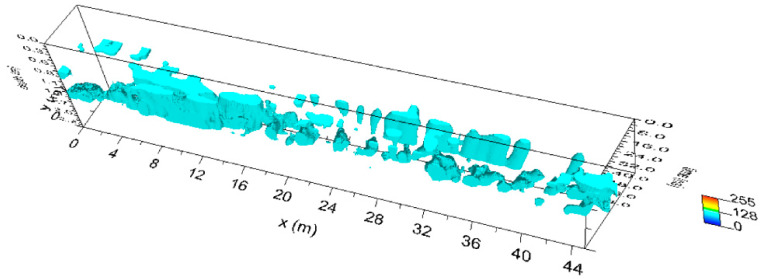
Plaza de Armas: sector A: three-dimensional amplitude for 600 MHz antenna.

Figure 13 show the three-dimensional amplitude isosurface using the 50% threshold.

In sector A the 200 MHz antenna results do not show results other than those obtained with the 600 MHz antenna.

### 5.4. Plaza de Armas Sector B

Sector B is adjacent to an area used for archaeological excavations which unearthed walls 50 to 90 cm thick of typical Inca ashlar masonry, composed of finely dressed granite blocks laid in regular courses, with an internal core of rubble masonry, and not laid in regular courses.

**Figure 14:**
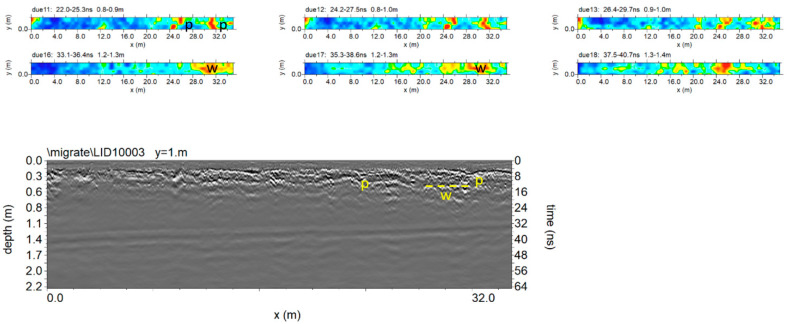
The 600 MHz processed radar section and time slice.

**Figure 15:**
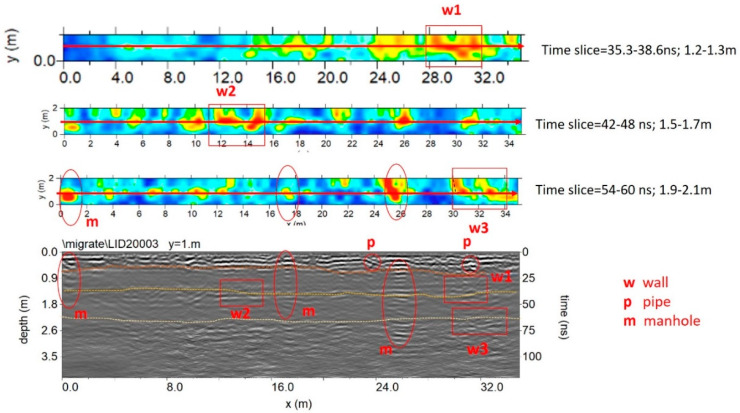
The 200 MHz processed radar section and time slice at 1.2 to 2.0 m exhibit a number of reflectors and high amplitude values considered to be to walls, shallow pipes and manholes.

Figure 14 and Figure 15 show the trace reflections and time slice for 600 MHz and 200 MHz antenna, respectively. The radargrams show at least three continuous reflectors which could be related to three different building phases and human frequentation. The shallowest is 50–60 cm deep, associated with two pipes (indicated as p in Figure 14 and Figure 15). The intermediate reflector is around 1.50 deep, related to a layer which includes two local reflectors (w1 and w2) which are considered to be walls. Finally, the deeper one is around 1.9–2.1 m deep, and is related to the oldest layer which includes a local reflector named w3, which is also considered to be a wall. Finally, the radargrams show repeated reflectors considered to be manholes (named ‘m’ in Figure 15).

## 6. Conclusions

**Figure 16:**
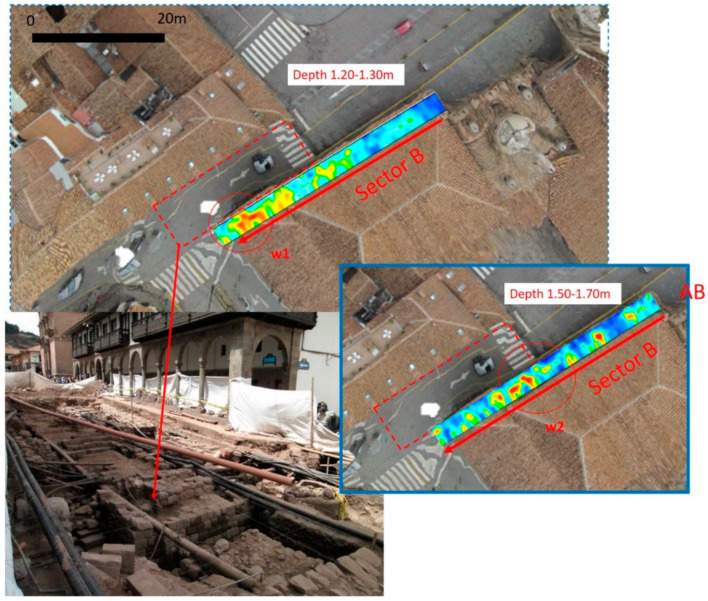
Comparison between the GPR and excavation results.

For the first time, geophysical prospection has been performed in Cusco, which within its territory, conserves the greatest number of architectural monuments and archaeological ruins dating to the Inca (having been the capital of Inca Empire; see Figure 1), and Colonial eras of South America. The case study shows the great potential of the GPR method in imaging complex stratigraphy up to 2 m deep, characterized by ancient walls, aqueducts, sewer pipelines and other structures linked to underground services. The crucial issue has been the interpretation of these diverse features. On the basis of direct data observed from some archaeological excavations, a model of the anthropogenic stratigraphy of Cusco has been assumed in order to simulate the wave reflection from walls and pipes. This has allowed us to conduct a morphological analysis of georadar features which have been compared with high amplitude values from horizontal time slices and three-dimensional amplitude visualization facilitating their interpretation. The center of Plaza de Armas, the main square of Cusco, and the southeastern arcade of the same square have been investigated. The interpretation of GPR results show the presence of walls, pipelines and manholes in the square and along the arcades. In particular, along the arcades of the square, GPR shows three different layers, among which, two are related to two anthropogenic layers associated with potential structures. These potential structures are in reasonable relation with walls unearthed by archaeologists in Calle Manta (Figure 16), and fit well with archaeological records, including pre-Inca ceramic fragments. This important result suggests the presence of three anthropogenic layers: pre-Inca, Inca and colonial, thus corroborating the hypothesis that suggests that the main square is the result of multiple phases of human frequentation (Aucca &-Caballero 2018; Cieza de Leon 1554; Vega 1609)

In the future, a geophysical-integrated approach [26] including georadar with lower frequency antenna and ERT will be adopted to explore in greater depth the urban soil in order to detect ancient urban transformation above the channelization of the Salphy and Tullumayo Rivers where Cusco was founded in the pre-Inca era.

## Figures and Tables

**Table 1:** Fresnel zone diameter at several depths (mean velocity 0.07 m/ns).

**200 MHz**	**Depth (m)**	0.5	1.0	1.5	2.0	2.5	3.0
	**Fz (m)**	0.62	0.85	1.04	1.20	1.33	1.45
**600 MHz**	**Depth (m)**	0.5	1.0	1.5	2.0	2.5	3.0
	**Fz (m)**	0.35	0.49	0.59	0.69	0.76	0.84
